# High Mean Platelet Volume Associates with In-Hospital Mortality in Severe Pneumonia Patients

**DOI:** 10.1155/2020/8720535

**Published:** 2020-06-08

**Authors:** Jieru Chen, Yihao Li, Yingsi Zeng, Yu Tian, Yueqiang Wen, Zebin Wang

**Affiliations:** ^1^Department of Intensive Care Unit, The Second Affiliated Hospital, Guangzhou Medical University, Guangzhou, China; ^2^Department of Anesthesiology, The Second Affiliated Hospital, Guangzhou Medical University, Guangzhou, China; ^3^Department of Nephrology, The Second Affiliated Hospital, Guangzhou Medical University, Guangzhou, China

## Abstract

**Background:**

Although mean platelet volume (MPV) appears to be associated with poor outcome of pneumonia, the relationship between MPV and in-hospital mortality is unclear in severe pneumonia (SP) patients.

**Methods:**

In this retrospective cohort study, 115 SP patients from June 1st, 2016, to September 29th, 2019, were included and divided into two groups. The primary outcome was in-hospital mortality. The receiver operating characteristic (ROC) curve was performed to assess the predictive ability for in-hospital mortality. Kaplan-Meier cumulative incidence curves were applied to observe the incidence of mortality. Multivariable Cox regression analyses were used to evaluate the hazard ratios (HRs). Besides, a formal test for interaction was investigated to analyze the relationship between MPV and sex.

**Results:**

During the course of hospitalization, 63 cases of mortality were recorded. ROC analysis suggested that MPV had a modest power for predicting in-hospital mortality (AUC = 0.723, 95% CI: 0.628-0.818, *P* < 0.001). Yet the cutoff value of MPV was 10.5 (sensitivity = 73.02%; specificity = 73.08%). Compared to the low-MPV group, the high-MPV group had significantly increased in-hospital mortality (log-rank test = 13.501, *P* < 0.001), while the adjusted Cox model indicated that the high-MPV group was associated with an elevated risk of in-hospital mortality (HR: 2.267, 95% CI: 1.166-4.406, *P* = 0.016). Moreover, analyses of in-hospital mortality suggested a significant interaction between optimal MPV level and sex (*P* = 0.011). In a multivariate Cox model which included females only, a high MPV level was associated with increased risk of in-hospital mortality (HR: 11.387, 95% CI: 1.767-73.380, *P* = 0.011).

**Conclusion:**

High MPV level is an independent risk factor for in-hospital mortality in patients with SP.

## 1. Introduction

Despite advances in its management over the last few decades, severe pneumonia (SP) remains a primary cause of death from infection across the globe, with a mortality rate ranging from 20% to more than 50% [1, 2]. Patients with SP are seriously ill and will progress to shock and even multiple organ dysfunction quickly if not treated properly in time [3]. Therefore, early prediction of mortality and intervention are of great importance for improving the prognosis of patients. However, information indicating risk factors for the development of death in SP patients remains sparse.

Traditionally considered as a hemostatic and thrombotic factor, platelet now has got increasing concentration for its role in inflammation and immunity [4]. It has been proven that platelet function and structure can alter markedly when reacting to physiological and pathological signals [5–8]. Mean platelet volume (MPV), a routinely measured marker in clinical practice, is a readily available and accurate indicator of platelet size and activity [9, 10]. Numerous studies have demonstrated that the alteration of MPV levels is correlated with the prognosis of infectious diseases. However, the evidence in pneumonia now is limited and controversial. Two studies showed that increasing MPV may be linked to poor outcomes [11, 12], whereas one study conversely showed a decrease [13]. Besides, there is an important difference in platelet biology impacted by sex. It has been reported that the sexual diversity in platelet sensitivity may be intrinsic to the platelet [14], and females showed a higher platelet reactivity than males [15, 16]. This feature suggests that the prognostic value of MPV in SP patients may be differed by sex. Nevertheless, no published research has discussed the influence of sex on the relationship between MPV level and mortality in SP patients.

Considering the abovementioned discrepancies, we compared demographic, clinical, laboratory, and radiological data and in-hospital mortality between groups of different MPV levels. Our objectives were to investigate whether MPV was associated with in-hospital mortality in SP patients.

## 2. Methods

### 2.1. Patient Population

In this retrospective research, we reviewed the electronic medical documents of consecutive adult patients with SP in the intensive care unit (ICU) of the Second Affiliated Hospital of Guangzhou Medical University from June 1st, 2016, to September 29th, 2019. Patients were included if they were admitted or transferred to the ICU with SP. The exclusion criteria were as follows: (1) younger than 18 years; (2) hospitalization in the ICU for less than 48 h; (3) hematological disorder; (4) advanced tumor; and (5) immunosuppressive diseases or receiving immunosuppressive therapy. This study protocol was approved by the Ethics Committee of the Second Affiliated Hospital of Guangzhou Medical University. Written informed consent was waived for the retrospective nature.

## 3. Definitions

SP was defined according to the expert consensus on clinical practice of SP in China (2016 Version). Pneumonia was defined as the presence of at least one of the first four items plus item 5: (1) new cough with or without sputum production or the original respiratory symptoms worsened, with purulent sputum and with or without chest pain; (2) fever; (3) pulmonary consolidation signs and/or moist rale; (4) peripheral blood leucocyte > 10 × 10^9^/L or <4 × 10^9^/L with or without a nuclear shift to the left; and (5) presence of a new chest radiographic infiltrate with or without pleural effusion and no clear evidence for alternative diagnoses. In patients with pneumonia, SP was diagnosed if patients met one of the major criteria or 3 of the minor criteria. The major criteria are (1) invasive mechanical ventilation and (2) septic shock with the need for vasopressors. The minor criteria are (1) respiratory rate ≥ 30 breaths/min; (2) PaO2/FiO2 ratio ≤ 250; (3) multilobar infiltrates; (4) confusion/disorientation; (5) uremia (BUN level > 20 mg/dL); (6) leukopenia (WBC count < 4 × 10^9^/L); (7) thrombocytopenia (platelet count < 100 × 10^9^/L); (8) hypothermia (core temperature < 36°C); and (9) hypotension requiring aggressive fluid resuscitation. The severity of illness was evaluated by acute physiology and chronic health evaluation II (APACHE II).

### 3.1. Data Collection and Outcome Measurements

Baseline demographic, clinical, and laboratory data were collected at admission to ICU. Baseline demographic data included age, sex, and underlying diseases (hypertension, diabetes mellitus, coronary heart disease, stroke, COPD, chronic kidney disease, and chronic liver disease). Clinical and laboratory data included hospital-acquired pneumonia, radiological findings (bilateral pneumonia, >2-zone involvement, and pleural effusion), interventions (mechanical ventilation, vasopressor use, and continuous renal replacement therapy), length of stay (ICU and hospital), mean arterial pressure, heart rate, respiratory rate, body temperature, APACHE II score, total bilirubin, albumin, serum urea, serum creatinine, red blood cell, hematocrit, hemoglobin, white blood cell, neutrophil count, lymphocyte count, platelet count, and platelet distribution width. Samples of peripheral blood were collected into tubes with ethylenediamine tetraacetic acid. MPV was determined using an automated blood cell analyzer (XN-2000, Sysmex, Japan). The primary outcome of this study was in-hospital mortality.

### 3.2. Statistical Analysis

Patients were divided into two groups (MPV ≤ 10.5 group and MPV > 10.5 group) according to the optimal value of MPV for predicting in-hospital mortality. The receiver operating characteristic (ROC) curve method was performed to assess the predictive ability of MPV for in-hospital mortality and to acquire MPV cutoff values to maximize sensitivity and specificity. Mean ± standard deviations were used to summarize normally distributed variables, medians (25th-75th percentile) were used to summarize skewed continuous data, and numbers and percentages were used to summarize categorical data. Independent two-group comparisons were performed using the Student *t*-test or Mann-Whitney *U* test for continuous data and chi-square test or Fisher's exact test for categorical data. Cumulative survival curves were constructed by the Kaplan-Meier method, and discrepancies between groups were evaluated by the log-rank test. A univariate Cox regression analysis was conducted to examine the correlation between patients' characteristics and in-hospital mortality. Furthermore, the multivariate Cox regression was conducted to explore variables significantly associated with the primary outcome, which adjusted for covariates (*P* < 0.1 in univariate Cox analysis or for importance of clinical concern (sex, COPD)). In addition, the interaction between MPV levels and sex was tested by performing a formal test of interaction. In Cox models, time at risk was from study entry until death during hospitalization, transfer, or discharge. According to the data distribution, missing covariate values were filled by mean or median. Statistical analyses were performed using SPSS, version 25.0, and *P* < 0.05 was considered significant.

## 4. Results

### 4.1. Baseline Characteristics

**Figure 1:**
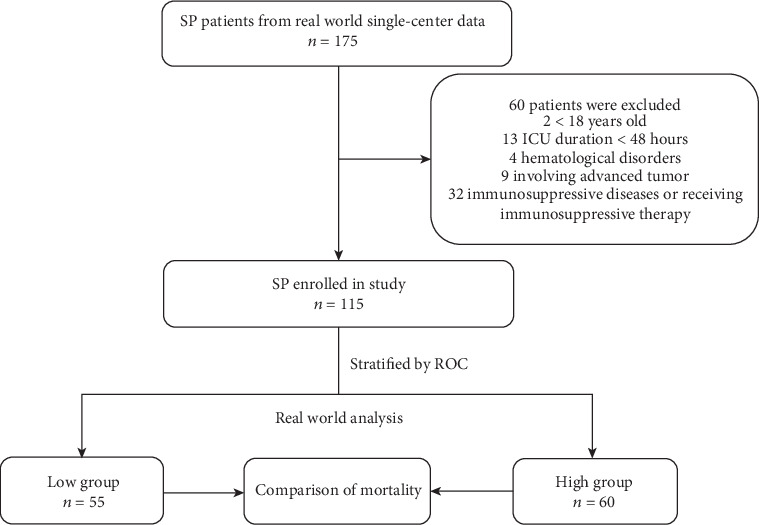
Study algorithm, including patient enrollment and outcomes. Note: low group: MPV ≤ 10.5; high group: MPV > 10.5. SP: severe pneumonia; ROC: receiver operating characteristic curve.

Of 175 patients with SP admitted to the ICU from 1st June 2016 to 29th September 2019, 115 patients were eventually enrolled in the study (Figure 1). Baseline characteristics of the cohort, categorized based on the optimal cutoff of MPV for predicting in-hospital mortality, were summarized in Table 1. The median patient age was 79 (63, 84), and 75 (65.2%) were male. The median APACHE II score was 20 (15, 24).

During follow-up, 63 (54.8%) patients died during the course of their hospitalization. A history of coronary heart disease and chronic pulmonary disease was significantly different between two groups (*P* = 0.049 and *P* = 0.027, respectively). Moreover, compared to the MPV ≤ 10.5 group, patients in the MPV > 10.5 group required more invasive mechanical ventilation and renal replacement therapy and had higher in-hospital mortality as well as APACHE II score (*P* = 0.034, *P* = 0.003, *P* < 0.001, and *P* = 0.005, respectively).

### 4.2. Performance of Baseline MPV as a Predictor of In-Hospital Mortality by ROC Curve Analysis

**Figure 2:**
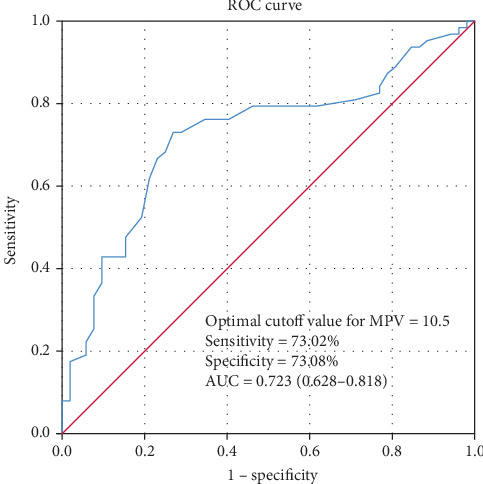
ROC curves of MPV for predicting mortality in patients with SP. MPV had a modest power for predicting in-hospital mortality as suggested by AUC of 0.723 (95% CI: 0.628-0.818, *P* < 0.001), with a sensitivity of 73.02% and a specificity of 73.08% at a cutoff of 10.5.

The ROC analysis showed that MPV had a modest power for predicting in-hospital mortality (AUC = 0.723, 95% CI: 0.628-0.818, *P* < 0.001), with a sensitivity of 73.02% and a specificity of 73.08% at a cutoff of 10.5 (Figure 2).

### 4.3. Risk Factors for Higher In-Hospital Mortality in SP Patients

The relevant risk factors for SP patients with higher in-hospital mortality were given in Table 2. After adjusting for covariates (*P* < 0.1 in univariate Cox regression and for importance of clinical concern (sex, COPD)), the multivariate analysis showed that COPD, vasopressor use, CRRT, and APACHE II score were associated with in-hospital mortality in patients with SP (*P* = 0.044, *P* = 0.048, *P* = 0.048, and *P* = 0.003, respectively).

### 4.4. MPV Associated with In-Hospital Mortality in SP Patients

**Figure 3:**
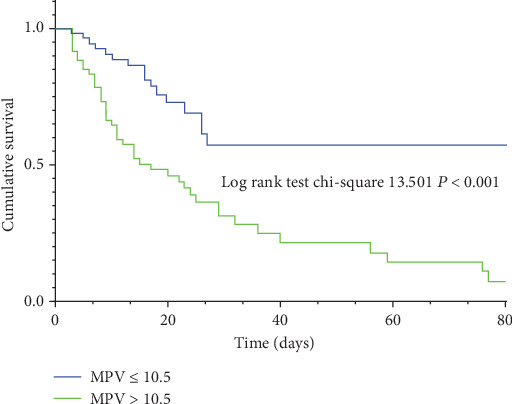
Kaplan-Meier survival curve according to MPV level above and below the optimal cutoff value (10.50 fL) for in-hospital mortality. Compared to the lower group (MPV ≤ 10.5), patients in the higher group (MPV > 10.5) showed elevated in-hospital mortality.

Kaplan-Meier survival curves for the MPV level according to the cutoff value were shown (Figure 3). Patients with MPV levels above the cutoff value had obviously higher mortality rates than those below the cutoff during the course of their hospitalization (log-rank test = 13.501, *P* < 0.001).

Associations of MPV with in-hospital mortality with defined models (with the MPV ≤ 10.5 group as the reference group) are given in Table 3. After adjustment, multivariate Cox analyses indicated that the MPV > 10.5 group was significantly associated with higher in-hospital mortality. In model 3, which was a maximally adjusted model containing age, sex, coronary heart disease, COPD, APACHE II score, mechanical ventilation, vasopressor use, CRRT, albumin, serum creatinine, and serum urea, adjusted HR for in-hospital mortality was 2.267 (95% CI: 1.166-4.406, *P* = 0.016).

### 4.5. Relationship between Mortality and MPV in the Sex Subgroup

A further study was conducted for the interaction between optimal MPV level and sex (Table 4). The results showed that there was a significant interaction between MPV level and sex on in-hospital mortality in SP patients (*β* = 2.075, *P* = 0.011). Therefore, a sex-stratified analysis was conducted using Cox models after adjustment for age, coronary heart disease, COPD, APACHE II score, mechanical ventilation, vasopressor use, CRRT, albumin, serum creatinine, and serum urea. In the female subgroup, there was a positive relationship between MPV and in-hospital mortality (HR: 11.387, 95% CI: 1.767-73.380, *P* = 0.011). In contrast, no relationship was observed in the male subgroup (*P* = 0.509).

**Figure 4:**
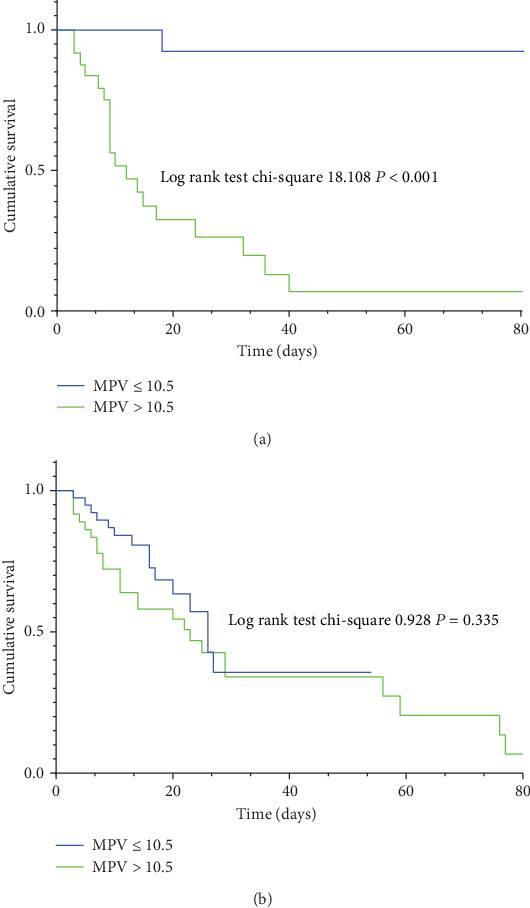
Cumulative survival curves for in-hospital mortality by category of MPV in the female group (a) and male group (b). Compared to the lower group (MPV ≤ 10.5), patients in the higher group (MPV > 10.5) showed elevated in-hospital mortality in female SP patients.

The Kaplan-Meier curve suggested that in the female subgroup, patients with MPV > 10.5 had higher in-hospital mortality rates than those with MPV ≤ 10.5 (log-rank test = 18.108, *P* < 0.001) (Figure 4(a)). Nevertheless, in the male subgroup, no significant difference was observed (log-rank test = 0.928, *P* = 0.335) (Figure 4(b)).

## 5. Discussion

In this study, we indicated that the MPV at ICU admission is positively associated with in-hospital mortality in female patients with SP, after adjusting for potential confounders. Meanwhile, no association was observed in the male subgroup. It may be the first time that the MPV has been discovered to be correlated with mortality in SP patients of different sex.

The prognostic value of MPV for mortality has been reported in severe infection. Gao et al. found that among platelet indices, the MPV had the highest prognostic ability for in-hospital mortality in septic shock patients [6]. Interestingly, they also found that the optimal MPV cutoff value for in-hospital mortality was at 10.5. Aydemir et al. reported a strong correlation between fungal sepsis and MPV [5]. Similar to prior studies, Kim et al. found that an increasing MPV was an independent risk factor for poor outcomes in patients with severe sepsis or septic shock [7]. Thus, we inferred that there was a potential relationship between MPV and the prognosis of SP. However, few studies have demonstrated the association between MPV and outcome in patients with SP.

Before this study, the relationship between MPV and in-hospital mortality was little investigated in patients with pneumonia. In one study containing 174 adults with community-acquired pneumonia (CAP), the MPV level below 8.55 fL was an independent risk factor for 28-day mortality [13]. In another investigation of 976 adults hospitalized for CAP, the results indicated that elevated MPV levels (MPV on discharge minus admission) may be related to in-hospital mortality [12]. These two studies only investigated patients with CAP. However, our study included both community- and hospital-acquired pneumonia, and patients in our study were much more serious. In addition, a retrospective study also suggested that the elevated MPV was related to poor outcome [11]. From day 2 onward, the MPV markedly increased in the nonsurvivor group, but no significant association between the MPV at admission and in-hospital mortality was found. This difference from our results may owe to the discrepancies in demography, baseline conditions, and adjusted confounders. The median age and MPV were relatively higher in our study (79 vs. 72.3 and 10.6 vs. 8.2, respectively). Moreover, radiological data not shown in their study, such as bilateral pneumonia and pleural effusion, may be associated with the poor outcome [17–19].

The underlying mechanisms of rising MPV with poor prognosis in SP patients remain hazy. We inferred that platelet activation played an important role in the progression of SP. Platelets are activated after exposure to platelet agonists, which are released from already activated platelets, impaired cells, or other inflammatory factors [20]. After activation, platelets take part in the immune system via various fashions, including direct interaction with pathogens, regulation of inflammatory responses by the congenital and complement immune system, and contact with leukocytes and specific immune system [20]. MPV is a measure of platelet size and a demonstrated indicator of platelet activation. Platelets with a higher MPV are larger and more reactive, indicating the existence of a thrombotic and inflammatory status [7]. Following the increased release of thrombopoietin and lots of inflammatory factors in serious infection, the platelet activation will be elevated and more large platelets will be released into the circulation [11, 21, 22]. Larger platelet expresses more procoagulant surface proteins and intracellular thromboxane A2 (TXA2), presenting a greater prothrombotic potency. TXA2 can also activate the pulmonary vascular endothelium, known to be integral in the process of acute lung injury related to serious infection [20]. In addition, platelet size has been reported to be a predictive marker of cardiovascular events [9]. Some evidence [23, 24] showed that platelet activation was related to acute coronary syndrome, and higher MPV was an independent risk factor for myocardial infarction in the pneumonia population. Besides, several studies reported that an elevated MPV was correlated to higher age, kidney dysfunction, and peripheral artery diseases [7, 12, 25]. Similarly, we also observed that a rise in the MPV was correlated to higher age and increasing serum urea and creatinine. Hypoxemia, which may lead to enhancive platelet consumption and bone marrow activation, is a potential explanation for the elevated MPV [12].

In particular, this study also found that a higher MPV level may be associated with higher in-hospital mortality in females. It may be the first time that the MPV has been found to be correlated with mortality in SP patients of different sex groups, which may suggest that the MPV is more likely to reflect the in-hospital mortality in female patients. The mechanism of this relationship is unclear, although sex differences in platelet function have been reported since 1975 [14]. Evidence demonstrated that compared to male patients, females have higher platelet counts and enhanced platelet activation [15, 16, 23, 26], which may be potential reasons. As discussed, platelet plays a pivotal role in inflammation via a variety of pathways. The increment of large platelets is related to the process of an inflammatory condition, possibly as a result of the intracellular synthesis of proinflammatory, procoagulatory factors, and platelet pool activation [9]. With elevated platelet activation and higher platelet count, platelets may release more cytokines and immunomodulatory ligands in females. Thus, female patients' platelets showed more attraction and interaction with neutrophils, leading to a greater inflammatory response. Moreover, higher activated platelets could result in disseminated intravascular coagulation (DIC) and embolism in the microvasculature, aggravating cell death and organ failure [20]. Platelets in females express a greater number of surface receptors, leading to binding much more fibrinogens and thus having a better ability to promote thrombosis and embolism [23]. Besides, one study including 3827 adults showed that a higher MPV was associated with metabolic disturbance only in females, which may be a potential contribution to higher mortality [27]. Anyway, the exact mechanism underlying the relationship between MPV and mortality in different sexes is poorly understood and should be extra studied.

The present investigation surely have limitations that need to be taken into consideration. First, this is a single-center retrospective study with a single racial population. Probable bias and residual confounders may have an influence on the results. Whether these findings could be applicable to other racial populations remains to be explored. Second, only the MPV at admission was included; whether repeated measurements during hospitalization would have imparted additional predictive information is uncertain. Third, the relatively low AUC of 0.723 suggested that MPV alone is not enough to predict mortality. The prognostic value of the combination of MPV and other prediction models for mortality needs to be further studied. Fourth, data of other inflammatory indices, such as C-reactive protein and procalcitonin, were missing; thus, the comparison of prognostic value between MPV and these indices remains unknown. Finally, the comparatively small sample size is a chief limitation of this investigation. Due to the small sample in this study, it is difficult to make an absolute conclusion, but as we observed a significant association between high MPV and higher in-hospital mortality in females, we suggested that clinicians should note the possibility of higher risk of death in this situation. Further large-scale multicenter prospective studies are needed to confirm the correlation between MPV and SP.

In conclusion, our study suggested that a higher MPV at ICU admission was associated with elevated in-hospital mortality in female SP patients. MPV, as a simple, inexpensive, and widely available indicator, may be a potential for risk stratification in female SP patients.

## Figures and Tables

**Table 1:** Comparison of baseline characteristics.

Variables	Total (*n* = 115)	MPV ≤ 10.5 (*n* = 55)	MPV > 10.5 (*n* = 60)	*P* value
Demographic data				
Age (years)	79 (63, 84)	79 (58, 83)	80 (67, 86)	0.273
Sex (male)	75, 65.2%	39, 70.9%	36, 60.0%	0.220
HAP	20, 17.4%	11, 20.0%	9, 15.0%	0.480
Underlying diseases				
Hypertension	67, 58.3%	34, 61.8%	33, 55.0%	0.459
Diabetes mellitus	32, 27.8%	13, 23.6%	19, 31.7%	0.337
Coronary heart disease	16, 13.9%	4, 7.3%	12, 20.0%	0.049
Stroke	38, 33.0%	20, 36.4%	18, 30.0%	0.469
COPD	29, 25.2%	19, 34.5%	10, 16.7%	0.027
Chronic kidney disease	14, 12.2%	8, 14.5%	6, 10.0%	0.456
Chronic liver disease	7, 6.1%	3, 5.5%	4, 6.7%	1.000
Radiological findings				
Bilateral pneumonia	87, 75.7%	42, 76.4%	45, 75.0%	0.865
>2-zone involvement	71, 61.7%	33, 60.0%	38, 63.3%	0.713
Pleural effusion	37, 32.2%	18, 32.7%	19, 31.7%	0.903
Clinical outcomes				
Mechanical ventilation	78, 67.8%	32, 58.2%	46, 76.7%	0.034
Vasopressor use	45, 39.1%	18, 32.7%	27, 45.0%	0.178
CRRT	41, 35.7%	12, 21.8%	29, 48.3%	0.003
ICU LOS (days)	11 (7, 18)	13 (8, 18)	9 (5, 21)	0.317
Hospital LOS (days)	16 (9, 27)	18 (11, 28)	14 (8, 25)	0.083
In-hospital death	63, 54.8%	17, 30.9%	46, 76.7%	<0.001
Clinical data				
MAP (mmHg)	83 (71, 97)	82 (71, 102)	83 (72, 95)	0.906
Heart rate (rate/min)	110 ± 26	109 ± 26	112 ± 25	0.536
Respiratory rate (rate/min)	27 ± 8	28 ± 8	27 ± 7	0.371
Body temperature (°C)	37.4 ± 1.1	37.4 ± 1.2	37.5 ± 1.0	0.847
APACHE II score	20 (15, 24)	18 (15, 23)	23 (17, 27)	0.005
Laboratory results				
Total bilirubin (umol/L)	13.0 (9.0, 21.8)	10.8 (7.4, 17.1)	14.0 (10.2, 25.4)	0.011
Albumin (g/L)	28.7 (26.8, 32.0)	29.9 (28.0, 32.1)	28.2 (25.4, 31.1)	0.064
Serum urea (mmol/L)	11.1 (8.1, 17.6)	10.0 (6.3, 15.1)	12.7 (9.2, 19.7)	0.007
Serum creatinine (*μ*mol/L)	120.4 (83.3, 187.8)	96.9 (73.7, 136.3)	130.8 (92.0, 213.8)	0.005
RBC (10^12^/L)	3.7 ± 0.9	3.7 ± 0.9	3.7 ± 0.9	0.960
HCT (%)	33.1 ± 8.1	33.1 ± 8.0	33.1 ± 8.3	0.970
Hemoglobin (g/L)	109.0 ± 26.3	106.1 ± 24.7	111.7 ± 27.7	0.260
WBC (10^9^/L)	12.5 (8.3, 18.0)	14.1 (8.9, 18.9)	11.4 (8.1, 16.5)	0.064
Neutrophil count (10^9^/L)	11.0 (7.1, 15.0)	11.6 (8.0, 16.6)	9.6 (7.0, 13.9)	0.104
Lymphocyte count (10^9^/L)	0.7 (0.4, 1.0)	0.8 (0.4, 1.2)	0.7 (0.4, 0.8)	0.093
Platelet count (10^9^/L)	222 (147, 288)	246 (203, 321)	168 (113, 245)	<0.001
PDW (fL)	12.8 (11.0, 14.5)	11.1 (9.7, 12.7)	13.6 (12.8, 15.5)	<0.001

Data are mean ± standard or medians (25th-75th percentile) or number and percentage.

MPV: mean platelet volume; HAP: hospital-acquired pneumonia; COPD: chronic obstructive pulmonary disease; CRRT: continuous renal replacement therapy; MAP: mean arterial pressure; APACHE: acute physiology and chronic health evaluation; LOS: length of stay; RBC: red blood cell; HCT: hematocrit; WBC: white blood cell; PDW: platelet distribution width.

**Table 2:** Independent predictors of in-hospital mortality by univariate and multivariate Cox regression analysis.

Factors	HR (95% CI)	*P*
Univariate Cox analysis		
APACHE II score	1.098 (1.064-1.133)	<0.001
Vasopressor use	2.778 (1.670-4.622)	<0.001
Mechanical ventilation	2.228 (1.205-4.119)	0.011
CRRT	2.446 (1.473-4.062)	0.001
Coronary heart disease	1.810 (0.934-3.508)	0.079
Age	1.018 (0.999-1.038)	0.059
Albumin	0.935 (0.884-0.989)	0.019
Serum urea	1.042 (1.018-1.067)	<0.001
Serum creatinine	1.001 (1-1.002)	0.060
Multivariate Cox analysis^a^		
COPD	1.937 (1.017-3.688)	0.044
Vasopressor use	1.842 (1.005-3.373)	0.048
CRRT	1.956 (1.004-3.809)	0.048
APACHE II	1.074 (1.025-1.126)	0.003

^a^Covariates included in multivariate analysis: age, sex, coronary heart disease, COPD, APACHE II, mechanical ventilation, vasopressor use, CRRT, albumin, serum creatinine, and serum urea.

MPV: mean platelet volume; APACHE: acute physiology and chronic health evaluation; COPD: chronic obstructive pulmonary disease; CRRT: continuous renal replacement therapy; HR: hazard ratio; CI: confidence interval.

**Table 3:** Relationship between MPV level and in-hospital mortality.

	MPV > 10.5 group	
In-hospital mortality	HR (95% CI)	*P*
Unadjusted	2.722 (1.552-4.773)	<0.001
Model 1	2.543 (1.438-4.499)	0.001
Model 2	2.268 (1.205-4.271)	0.011
Model 3	2.267 (1.166-4.406)	0.016

Reference group is MPV ≤10.5 group.

Model 1: age and sex.

Model 2: model 1 plus comorbid conditions and clinical data (coronary heart disease, COPD, APACHE II score, mechanical ventilation, vasopressor use, and CRRT).

Model 3: model 2 plus albumin, serum creatinine, and serum urea.

MPV: mean platelet volume; COPD: chronic obstructive pulmonary disease; APACHE: acute physiology and chronic health evaluation; CRRT: continuous renal replacement therapy; HR: hazard ratio; CI: confidence interval.

**Table 4:** Relationship between in-hospital mortality and MPV level by sex.

In-hospital mortality	Male		Female		Interaction	
	HR (95% CI)	*P*	HR (95% CI)	*P*	*β*	*P*
MPV > 10.5^a^	1.392 (0.521-3.717)	0.509	11.387 (1.767-73.380)	0.011	2.075	0.011

^a^Adjusted for age, coronary heart disease, COPD, APACHE II, mechanical ventilation, vasopressor use, CRRT, albumin, serum creatinine, and serum urea.

MPV: mean platelet volume; COPD: chronic obstructive pulmonary disease; HR: hazard ratio; CI: confidence interval.

## Data Availability

The data used to support the findings of this study are included within the article.
